# An Update on Mitral Valve Aging

**DOI:** 10.3390/life14080950

**Published:** 2024-07-28

**Authors:** Carmen Elena Opris, Horatiu Suciu, Cosmin Ioan Opris, Simona Gurzu

**Affiliations:** 1 Department of Adult and Children Cardiovascular Recovery, Emergency Institute for Cardio-Vascular Diseases and Transplantation, 540139 Targu Mures, Romania; carmenchincisan@yahoo.com; 2 Department of Pathology, George Emil Palade University of Medicine, Pharmacy, Science, and Technology, 540139 Targu Mures, Romania; 3 Department of Surgery, George Emil Palade University of Medicine, Pharmacy, Science, and Technology, 540139 Targu Mures, Romania; horatiu.suciu@umfst.ro (H.S.); nymcos@yahoo.com (C.I.O.); 4 Romanian Academy of Medical Sciences, 030173 Bucharest, Romania; 5 Department of Cardiovascular Surgery, Emergency University Hospital, 050098 Bucharest, Romania; 6 Research Center for Oncopathology and Translational Medicine (CCOMT), George Emil Palade University of Medicine, Pharmacy, Science, and Technology, 540139 Targu Mures, Romania

**Keywords:** heart, mitral valve, aging

## Abstract

The aging process can have notable effects on the mitral valve. During life, the mitral valve undergoes various changes that can impact its structure and function. The purpose of this review is to present a comprehensive overview of the literature published up to February 2024 in the Medline database regarding the impact of aging on the mitral valve. The studies were combined with the personal experience of the authors. Until 2024, out of the 12,189 publications that deal with the mitral valve in elderly individuals, 308 refer to mitral valve aging. After reviewing these data, we selected and analyzed the 73 most informative works regarding the age-related transformation of the mitral valve. Understanding the mechanisms driving the aging of the mitral valve is crucial for enhancing diagnostic and therapeutic strategies for reducing age-related valve dysfunction and the subsequent cardiovascular complications.

## 1. Introduction

Both morphologically and functionally, the mitral valve represents a complex apparatus, consisting of the mitral valve in the form of the valvular leaflet (anterior and posterior) and the mitral valve annulus and subvalvular apparatus represented by the chordae tendineae and papillary muscles. Since mitral valve apparatus development is complete in the 15th week of intrauterine life [1,2], the function of the mitral valve is to permit blood to flow from the left atrium to the left ventricle during diastole and to prevent the retrograde passage of blood during systole. It also has an important role in the systolic function of the left ventricle [3]. The normal function of the mitral valve depends on the atrial wall and mitral annulus, as well as the mitral leaflets, chordae tendineae, papillary muscles, and ventricular myocardium. These act together in the normal function of the mitral valve [4].

The wall of the left atrium extends over the proximal portion of the posterior mitral leaflet, and thus, an increase in the size of the left atrium results in damage to the posterior leaflet with the onset of mitral regurgitation. In the case of a normal mitral valve, there is no continuation of the ventricular myocardium to the atrial wall [4]. The atrial and ventricular walls are separated by a fibrous area where the fibrous ring to which the mitral leaflets are attached is also located [5].

Mitral valve disease is the second most common cause of surgical interventions in cardiovascular surgery. Mitral valve regurgitation can be primary or secondary depending on the etiology. The causes of primary mitral valve disease include rheumatic disease, mitral valve prolapse and flap, infectious endocarditis, some drugs (anorectic drugs), and collagen vascular disease [6]. Secondary or functional mitral valve regurgitation is caused by the dilation, abnormal shape, and/or dysfunction of the left ventricle and/or dilation of the left atrium and mitral annulus with a normal mitral valve structure [7].

Aging of the valve causes degenerative changes in the mitral valvular apparatus, resulting in the appearance of mitral insufficiency or stenosis.

Repeated mechanical stress on the mitral valve leaflets can cause wear and tear over time. Thus, the histological structure of the valve changes, and it becomes less flexible. Fibrosis and calcification appearing over time are additional histological and biochemical changes at the level of the mitral valve. All of these have an impact on the normal functionality of the mitral valve over time, causing insufficiency or mitral stenosis [8].

Valvular changes are identified by echocardiography. The treatment of mitral valve disease is surgical or interventional, and the identification of valvular changes guides the surgeon in making the optimal therapeutic decision. If it is suitable, mitral valvuloplasty is always preferred to valve replacement with mechanical or tissue prosthesis. An alternative is interventional treatment through the transcatheter implantation of a clip at the level of the valvular edges. Determining the changes that occur with aging at the level of the mitral valve is important for making the right therapeutic decision.

This review aims to provide a thorough overview of the literature up to February 2024 from the Medline database on the impact of aging on the mitral valve. We have integrated these studies with our own experiences. By 2024, out of 12,189 publications concerning the mitral valve in elderly individuals, 308 specifically address mitral valve aging. From these, we have reviewed and analyzed the 73 most informative studies on the age-related transformations of the mitral valve.

## 2. Normal Structure of the Mitral Valve

### 2.1. Mitral Annulus

The mitral ring is a fibrous structure that forms the base of the mitral valve cusps. It is not complete and is slightly flattened and D-shaped, with the straightest part located toward the posterior aortic wall. This flattened part allows the aortic ring to fit between the mitral valve and the interventricular septum. The mitral ring is part of the fibrous skeleton of the heart. Under the microscope, this structure is composed of dense collagen fibers, with a few intercalated elastic fibers as well as areas of cartilage. The anterior leaflet, also named the aortic leaflet, is much smaller, occupying only one third of the valve’s circumference but is broader than the posterior/mural leaflet [9,10]. A deformity of the mitral ring causes mitral regurgitation [11,12].

### 2.2. Mitral Leaflets

Mitral leaflets were described in 1952 by Harken et al. as a continuous veil lining the mitral orifice. They have two commissures and several incisions on the valve edges [13]. The two commissures (anterolateral and posteromedial) delimit the two leaflets: anterior and posterior. The mitral valve is a supple structure covered by the endothelium. It is generally avascular, and its nutrition is achieved by diffusion. The normal thickness of a valve is 3–5 mm [14,15].

The normal mitral orifice area is 5–7 cm^2^, or, as Riggs et al. suggest, 4.2 cm^2^/m^2^ [13]. It is smaller in women than in men. The surface of the valve sheet is 14 cm^2^ [14]. The calculation formula proposed by Riggs et al., using the mitral valve area (MVA) in cm^2^ and the body surface area (BSA) in m^2^, is MVA = 4.83 × BSA − 0.07 [16].

The mitral ring has an ellipsoid shape that changes during the cardiac cycle. The maximum lateromedial diameter is approximately 30 mm [15].

The anterior mitral leaflet, which has a semicircular shape, is also called the aortic, anteromedial, larger, or septal valve [17]. The anterior mitral valve is inserted over an area of 45% of the mitral ring [15]. The description and mitral valve repair technique developed by Carpentier more than 50 years ago are still available.

The valvular leaflets have a base and free edge with a rough aspect on which the tendon cords are inserted. Between the two portions is a clear, translucent intermediate area without cord insertion. A prominent ridge (1 cm from the free edge) is located at the joint of the clear area with the rough zone. It is also present on the surface of the posterior leaflet, with the closing of the two valves occurring at this level [18].

**Figure 1:**
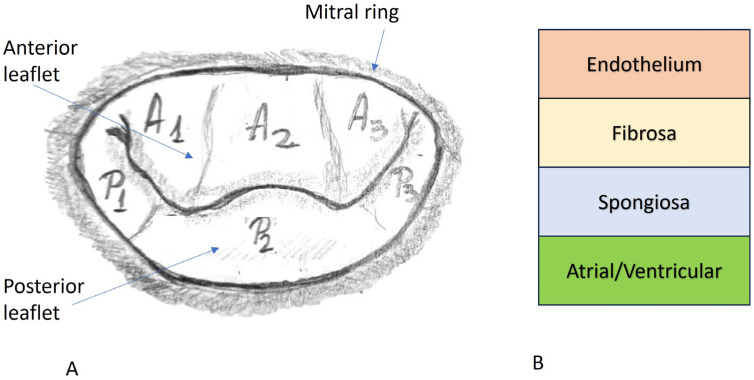
(**A**) Gross anatomy of the mitral valve. (**B**) Micro-anatomy of the mitral valve.

The posterior mitral leaflet, known as the ventricular, mural, or posterolateral leaflet, is smaller than the anterior one, has a semilunar shape, and is inserted on 55% of the circumference of the mitral ring. It is divided by incisions of the free edge into three segments: from lateral to medial: P1—near the anterolateral commissure, P2—situated centrally, with a variable shape and size, and P3—located posteromedial. These correspond to the scallops of the anterior valve A1, A2, and A3. The incisions that divide the valve edges do not reach the base of the valve [18] (Figure 1).

**Figure 2:**
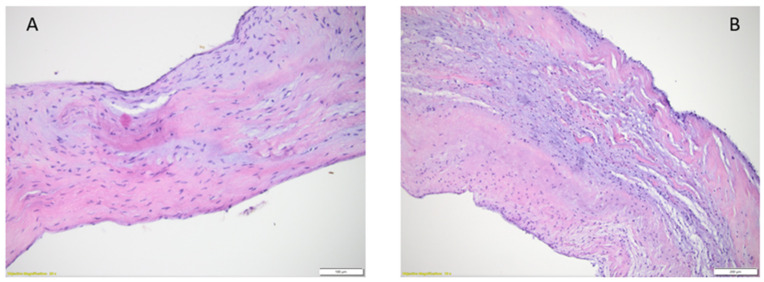
Histological aspect of the mitral leaflets. (**A**) adult mitral valve, objective magnification 20×, (**B**) child mitral valve, objective magnification 10×.

Histologically, the leaflet of the mitral valve comprises four layers. From the atrial to the ventricular surface of the valve, it is composed of the atrialis/auricularis, spongiosa, fibrosa, and ultimately, the ventricularis. The atrialis is composed predominantly of elastic fibers intermingled between collagen fibers and smooth muscle cells. Underneath the atrialis, the spongious layer is a well-represented, paucicellular layer composed of loose connective tissue with sparse elastic fibers and collagen fibers, rich in glycosaminoglycans (GAGs), predominantly chondroitin sulphates, heparin sulphates, and hyaluronic acid. The atrialis covers the spongious layer only at the level of the proximal one third of the valve, close to the fibrous ring (annulus fibrosus). At this level, the spongiosa may contain myocytes and capillaries as an extension from the vascular supply of the left atrium. Classically, the distal two thirds of the mitral valve are considered non-vascularized, but some recent studies have postulated that capillaries might appear distally within the structure of spongiosa in the absence of any known associated inflammatory condition. The best-represented layer is the fibrosa, which, unlike the others, follows the entire length of the mitral leaflet, from the annulus fibrosus until the free edge of the leaflet. It has a denser histoarchitecture, represented mainly by dense connective tissue oriented in a circumferential fashion, that bridges the fibrous ring and the apex of the papillary muscles. As the most well-represented layer with the densest structure, the fibrosa is responsible for the physical support of the valvular architecture. If the spongiosa has a precise role in tissue compressibility during every cardiac cycle, the fibrous layer is the valvular structure of resistance. Like the atrialis, the ventricularis only partially covers the ventricular aspect of the valve. This layer is composed predominantly of elastic fibers blended with loose collagen fibers and extends from the subendocardium of the left ventricle (Figure 2) [19].

### 2.3. Chordae Tendineae

The chordae tendineae are fibrous structures that attach to the ventricular face of the mitral valve leaflets. In general, the cords have three ends. One is inserted on the free edge and two in the rough area. Some authors describe atypical chordae that have fewer than three branches. The chordae that are entered on the posterior mitral leaflet are generally thicker, shorter, and stiffer than those on the anterior one.

On the anterior leaflet, there are two thicker cords. These are called “strut cords” and originate from the tip of the papillary muscles. The chordae tendineae can be primary, which are inserted at the free edge of the leaflets, or secondary, at the base of the leaflets. The cleft chordae enter into the indentations of the posterior leaflet [17]. Usually, the chordae tendineae start from the tip of the papillary muscles. There are also tertiary cords that begin from the ventricular myocardium and are introduced at any level of the leaflet surface, including the clear area for equal distribution of systolic stress. Fake chordae arising from the ventricular wall and attached on the ventricular wall have also been described [3,20,21,22]. Occasionally, a type of muscular cord can be seen with a thickness greater than 3 mm that is inserted in the aortic valve. These are considered benign and very rarely noted [23].

Knowing the insertion site of these strings plays a very important role in mitral valve surgery. It has been observed that the leaflets prolapsing in the left atrium, especially in the elderly, have an abnormal distribution of strings on the ventricular surface. In this case, the leaflets have less sustained and less resistant areas [23].

**Figure 3:**
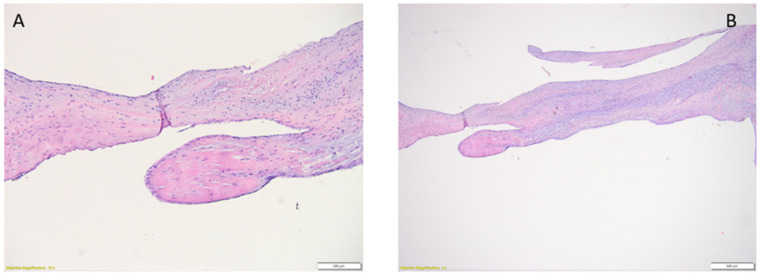
Histological aspect of the chordae tendineae. (**A**) adult mitral valve, objective magnification 20×, (**B**) child mitral valve, objective magnification 10×.

Chordae tendineae are composed of three different layers. Their core is represented mainly by dense collagen fibers oriented parallel to the long axis, a distribution that increases resistance. Besides the collagen bundles, the inner layer also contains fibroblasts and nerve fibers. The middle layer, called the subendothelial layer, is composed of elastic fibers, fibroblasts, and loose collagen fibers. Ultimately, the outer layer is represented by monostratified endothelial cells separated from the subendothelial layer by a basal lamina, which is composed of type IV collagen. At the junction between the chordate tendineae and papillary muscles, the fibrous core of the chordae tendineae arborises and intermingles its fascicles between papillary muscle myocytes (Figure 3) [24,25].

### 2.4. Papillary Muscles and the Ventricular Myocardium

The papillary muscles and the ventricular myocardium represent the muscular part of the mitral valve system. They are extremely important in the competence of the mitral valve. The papillary muscles are inserted at the apex and apical third of the left ventricular myocardium, with the anterolateral larger than the posteromedial one. Although they are classically described as two papillary muscles, they may be close muscle groups, fused at their base by muscular or even tendon bridges before insertion at the ventricular level [26]. From the tip of the papillary muscle arise the chordae tendineae that are connected to the anterior and posterior leaflets of the mitral valve. Both papillary muscles have connections to each of the leaflets for an equal distribution of systolic stress [3].

The thickness of the papillary muscle is approximately the same as the free wall of the left ventricle. Each muscle may vary, including in thickness and number. Usually there are two papillary muscles, but in a study performed by Saha and Roy, the number varied from two to seven [26]. Sometimes only one anterolateral papillary muscle can be identified [27].

Usually, the anterolateral papillary muscle has one head and the posterolateral papillary muscle has two. The morphology also might vary. The shape of papillary muscle has been described as conical, flattop, or bi- or trifurcated [26].

The contraction of the papillary muscles, which shortens in systole by up to 20%, approximately 4 mm, is simultaneous to maintain the valvular leaflet, as proved by the reduction of mitral regurgitation by cardiac resynchronization therapy during functional mitral regurgitation in a case of dilated cardiomyopathy with a major left branch block [3,28].

## 3. Age-Induced Histological Modifications of the Mitral Valve

The heart beats over 100,000 times a day, and the heart valves must withstand this permanent mechanical stress. The mitral valve opens in diastole in 50 ms, remains open for 600 ms, then closes in 50 ms and remains closed in systole for 300 ms [29]. This happens approximately 40 million times a year and over three billion times in a lifetime [30]. To resist, the extracellular matrix, which contains elastic fibers, proteoglycans, glycosaminoglycans, collagen fibers, growth factors, and signaling molecules, constantly adapts [31,32]. It has the role of both supporting the interstitial cells but also providing mechanical resistance in the form of biological signaling. Under the action of mechanical forces and biochemical signals, valve cells (interstitial and endothelial) respond to phenotypic changes by changing the structure of the extracellular matrix [33,34]. The interstitial cells are very sensitive to changes in the environment around them, influencing each other with the structure of the extracellular matrix [34]. In cases of increased mechanical strength and valve stress (e.g., hypertension, mitral valve prolapse), tissue adaptation occurs that leads to shifts in their structure and composition [35]. Alterations of valvular stress occur in physiological conditions and diseases but also in cases of valve surgery repair [36].

In young adults, both the atria and ventricles contain multiple thin layers of elastic fibers, with more in the atrium than in the ventricles. Some collagen fibers are also present. As the years pass, the annulus becomes thicker because of fat cell deposits, which can also be seen on the free edge of the leaflets. In addition, the collagen density in the fibrosa becomes more irregular and increases. After the age of 40 years, the collagen and elastic fibers become fragmented. The amounts of collagen and elastic fibers increase with age, in addition to the fragmentation degree, and reach the highest level after the age of 80 years, when the fibrosa is the thickest and the spongiosa the thinnest [37]. In aged valves there is a change in the type I and type III collagen ratio that affects the valves’ thickness and stiffness, with the same effect produced by the increase in the local synthesis of proteoglycans and glycosaminoglycans [38]. Myofibroblasts fail to maintain a balance between synthesis and connective tissue degradation, and thus, degenerative changes in the valves occur and worsen. Mitral ring calcification and valvular retraction may also be encountered, especially in patients with previous rheumatic disease [33,34]. Thus, the valves’ interstitial cells are the determinants of the myxomatous changes and calcifications that occur in aged valves [39,40].

Increasing amounts of data suggest a role for the extracellular matrix in the aging of the heart and the heart valves. Aging leads to a cascade of phenomena that cause structural and functional changes in the composition of the extracellular matrix of the heart valves [41].

In addition, the remodeling of the extracellular matrix itself modifies the secretory function of the interstitial and endothelial cells, contributing to valve degeneration [33]. This remodeling, both physiological and pathological in the context of diseases, causes the weakening of the fibrous layer of the valves, and substances secreted by interstitial cells can easily pass between the valve layers, thus creating a local inflammatory process that maintains and stimulates degenerative changes [33]. There are also adipocytes and lipid deposits. The latter are usually on the ventricular surface but may also appear on chordae tendineae [42]. The tips of the mitral valve leaflets may exhibit nodular thickening [43].

Increased mechanical load leads to excess collagen tissue while its decrease, such as in myxomatous disease or ischemic disorders, accumulates more proteoglycans [31,44]. The leaflets become less translucent. These modifications are also seen on the mitral valve annulus and are degenerative disease. Mitral ring calcification is described in half of individuals over 60 years in echocardiography, but its pathophysiology is incompletely understood [45,46,47]. It is very rarely found in people with the serum cholesterol level under 150 mg/dL [48]. These calcifications represent an independent risk factor for stroke (besides atrial fibrillation, congestive heart failure, or ischemic heart disease) [45,49]. Often, calcification of the posterior mitral annulus also encompasses the base of the posterior mitral leaflet protruding in the left ventricle cavity. The consequence might be mitral regurgitation or mitral stenosis [48]. Another complication of mitral annulus calcification is the extension of calcification in the conduction system leading to different degrees of heart block. Severe mitral annulus calcification can cause difficulties in surgical replacement of the mitral valve, leading to free ventricular wall rupture, injury to the circumflex artery during debridement, or para-prosthesis regurgitation [47].

## 4. Physiopathology of the Mitral Valve: Age-Induced Modifications

**Figure 4:**
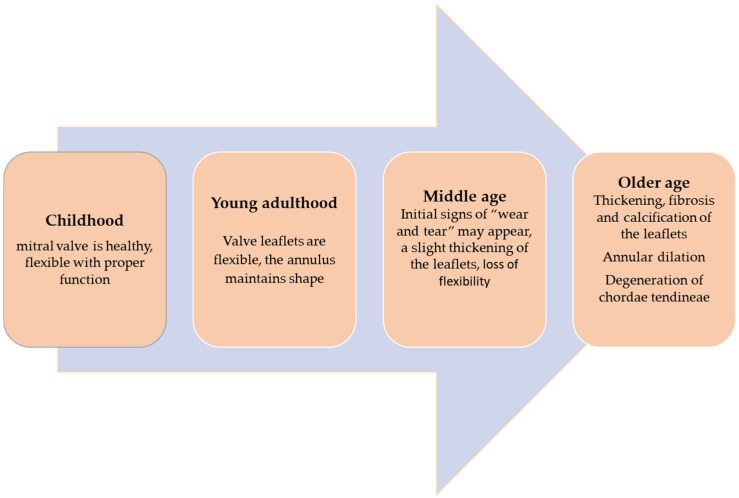
Changes in mitral valve over time.

The mitral valve plays a role in the unidirectional conduction of blood flow from the left atrium to the left ventricle during the diastolic phase of the cardiac cycle. Diastole has four phases: the isovolumic relaxation phase, rapid filling, diastasis, and atrial contraction. During the first phase the mitral valve is closed, and the ventricular pressure drops under the intra-atrial pressure. In the next phase, due to ventricular suction, a rapid filling occurs, which provides 70–80% of the ventricular filling. Next, a stage of equalization of atrio-ventricular pressures (diastase) follows, and approximately 5% of the ventricular filling is completed. The atrial systole will raise the atrioventricular gradient, allowing 20–30% of the ventricular filling in end-diastole. Diastole ends when the atrial and ventricular pressures equalize, and the mitral valve closes [50,51,52,53]. The evolution of the mitral valve over time is presented in Figure 4.

The mechanical forces exerted on the mitral valve can significantly influence its aging process. The mitral valve experiences constant mechanical stresses due to the cyclic opening and closing of the valve leaflets during each cardiac cycle. These mechanical forces can lead to structural changes in the valve over time and play a crucial role in the aging of the mitral valve by promoting wear and tear, fibrosis, calcification, and remodeling of the valve leaflets and supporting structures.

The forces acting on the mitral valve during a cardiac cycle include flexion through the closing and opening movement, friction due to blood flow, and tension from closing the valve to prevent retrograde blood flow [53]. The repetitive mechanical stresses on the mitral valve leaflets can cause “wear and tear” over time [54,55]. This can lead to thinning of the valve leaflets, a loss of flexibility, and alterations in the extracellular matrix composition.

Other mechanisms through which mechanical forces contribute to the aging of the valve include fibrosis and calcification. Fibrosis serves as a fundamental biological response aimed at repairing damaged tissues, especially when the body’s natural regenerative capabilities are insufficient to fully restore them.

**Figure 5:**
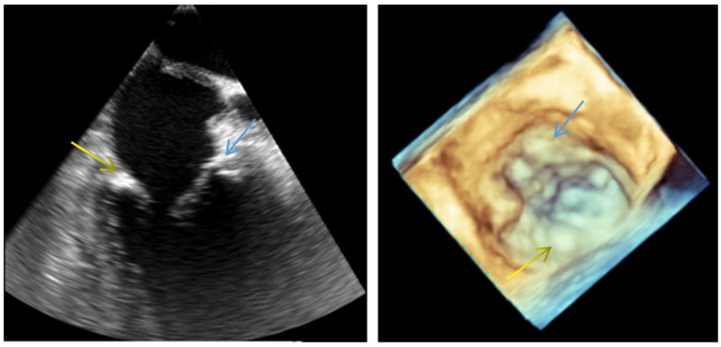
Transesophageal 2D and 3D echocardiography showing severe mitral annulus calcification. Blue arrow: anterior side of the mitral ring; yellow arrow: posterior side of the mitral ring.

Fibrosis involves the excessive deposition of collagen and other extracellular matrix proteins, which can stiffen the valve leaflets and impair their function [56]. Furthermore, mechanical stresses can also contribute to the calcification of the mitral valve, a process in which calcium deposits accumulate within the valve leaflets. Calcification further stiffens the valve and impairs its ability to open and close properly. The diagnosis is confirmed by echocardiography (Figure 5).

Individuals aged over 75 years old experience annual incidental rates of mitral ring calcification of approximately 4.7%, contrasting with younger patients under 55 years old, who are comparatively less affected by such occurrences [57].

In a study involving patients aged 72–91 years, with no history of heart disease, researchers observed the typical calcification primarily around the annulus or the points where the chordae tendineae attach to the valve. However, they noted minimal alterations in the mechanical properties of the valve leaflets themselves [58].

Mechanical forces not only act directly on the valve leaflets but also affect the supporting structures of the mitral valve, such as the chordae tendineae and the papillary muscles. Chronic mechanical stress can lead to remodeling of these structures, including the elongation, thickening, or rupture of chordae tendineae, which can further compromise valve function and contribute to the aging process.

## 5. Discussion

During life, the mitral valve closes and opens almost three billion times [29]. The aging of the valve determines the thickening of the valve leaflets, especially by increasing the thickness of the fibrous layer, with an increase in the number of collagen fibers and the degree of fragmentation of collagen and elastic fibers. Moreover, the mechanical forces that normally act on the mitral valve determine changes in the extracellular matrix. Fibrosis and calcification of the valve leaflets and the mitral annulus over time also lead to the progression of mitral valve disease. The changes are found both at the level of the valves and the tendinous chordae.

Valvular stress occurring under normal conditions, but especially in the case of changes in valve geometry, both in valve diseases and mitral valvuloplasty, determines tissue adaptation through changes in the structure of the extracellular matrix. At this level, the remodeling of collagen fibers, a reduction in elastin, and the alteration of the synthesis of glycosaminoglycans and proteoglycans occur. Collagen fibers become more irregular and denser over the years. Similar changes can be seen in the elastic fibers, which become granular. Additionally, the fragmentation of collagen and elastic fibers increases with age. The interstitial cells are responsible for the biochemical changes inside the valves, with an increase in proteoglycans in the fibrous layer. All these changes are much more pronounced in patients with myxomatous degenerative mitral disease [59].

Mild or moderate mitral valve regurgitation is a normal finding in asymptomatic patients, and its incidence increases with age [60].

Aging of the mitral valve is a complex process involving changes at different levels of the mitral valve apparatus. The aggravation of mitral valve damage in a patient is multifactorial, and degeneration due to aging has an important role in the evolution of the disease. Chronic mitral valve regurgitation advances at an average rate of 7.4 mL of increased blood backflow per year. Over time, this can cause the left ventricle to remodel, altering its size, shape, and function, which can lead to ventricular dysfunction and, eventually, heart failure.

The progression of mitral regurgitation is greater if an intrinsic valve disease is present, such as myxomatous degenerative disease with mitral valve prolapse. Additionally, the initial degree of mitral regurgitation and the size of the ring are important for assessing the evolution of the disease: a higher degree of regurgitation and a dilated ring evolve faster toward the worsening of the disease [61].

Echocardiographic changes related to heart aging were studied. It is well known that aging does not affect the left ventricle cavity diameter, the fractional shortening of the minor semi-axis, and the velocity of circumferential fiber shortening. However, aging is associated with altered diastolic filling, left ventricle hypertrophy, and increased aortic root dimension [62]. There is a significant increase in heart weight due to hypertrophy of the septal and lateral walls of the left ventricle and valve circumferences. The coronary arteries are more calcified, dilated, and tortuous. Regarding the changes seen in mitral leaflets, they thicken and become fibrotic along their edges. In the mitral valve annulus, sites of collagen degeneration, lipid accumulation, and calcification are present [63]. Degeneration of the conduction system of the heart also occurs, with the conduction disturbance more often seen in older people. The normal sinus rate and escape rates of other automatic cells slow down. Sensitivity to cardiac drugs is also affected [64].

The aging process of the mitral valve is influenced by various molecular pathways that contribute to structural and functional changes [65]. In the mechanism of aging, an important role is attributed to the renin–angiotensin System (RAS). Angiotensin II induces inflammation by stimulating the release of inflammatory cytokines. It also promotes fibrosis and calcification through increased production of reactive oxygen species (ROS) and up-regulation of pro-fibrotic factors like transforming growth factor-beta (TGF-β). [66].

**Figure 6:**
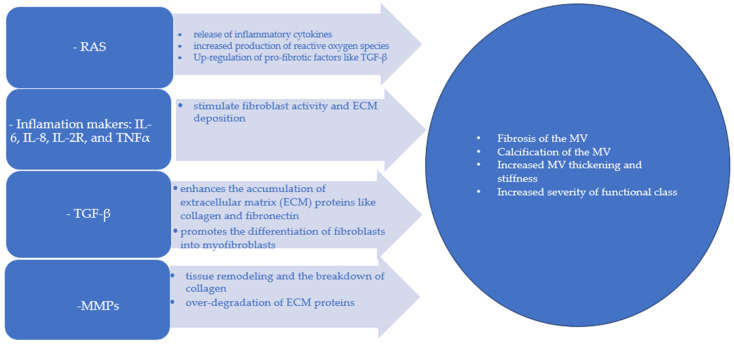
Bio-sketch of the molecular pathways involved in the aging of valves. RS: renin–angiotensin system, IL-6: interleukin-6; IL-8: interleukin-8, IL-2R: interleukin-2 receptor; TNFα: tumor necrosis factor α, MMPs: matrix metalloproteinases, MV: mitral valve.

Patients with rheumatic heart disease exhibit elevated levels of circulating interleukin-6 (IL-6); IL-8, IL-2 receptor (IL-2R) and tumor necrosis factor α (TNFα). Additionally, IL-6 and TNFα levels were associated with increased valve calcification and greater severity of functional class [67]. TGF-β signaling enhances the accumulation of extracellular matrix (ECM) proteins like collagen and fibronectin, leading to fibrosis. It also promotes the differentiation of fibroblasts into myofibroblasts, which are cells that play a key role in ECM production and remodeling of the mitral valve [68]. In terms of ECM reorganization, there is often a marked increase in the hyaluronic acid content along with a reduction in sulfated glycosaminoglycans. This change is linked to the TGF-β-mediated downregulation of genes associated with A disintegrin and metalloproteinases, which are crucial for proteoglycan degradation. As a result, the mechanical properties of myxomatous mitral valve leaflets are altered, leading to greater valve extensibility, reduced leaflet stiffness, and lower failure strain. Matrix metalloproteinases (MMPs) play a crucial role in tissue remodeling and the breakdown of collagen. Imbalanced MMP activity results in the over-degradation of ECM proteins, compromising the structural integrity of the mitral valve. This disruption between ECM production and breakdown leads to valve thickening and stiffness. Initially recognized for their crucial roles in tissue morphogenesis and wound healing, MMPs have also been found to be involved in the intricate remodeling processes of blood vessels and the myocardium [69] (Figure 6).

The oxidative stress theory of aging states that functional declines associated with aging result from the accumulation of damage caused by reactive oxygen and nitrogen species (RONS). These findings are important for future therapies used for slowing the evolution of aging of mitral valve disease [70].

Reducing age-induced changes of the mitral valve is an important step in preventing mitral valve disease. First, lifestyle changes such as a healthy diet rich in antioxidants, vitamins, and minerals, regular physical activity, and quitting smoking can lower oxidative stress and inflammation and may slow valve degeneration. Drugs such as ACE inhibitors and ARBs reduce inflammation and fibrosis trough the reduction of IL-6 and TNF-α [71]. Other drugs that can slow the evolution of mitral valve degeneration are statins. Besides lowering cholesterol, statins have anti-fibrotic and anti-inflammatory effects [72]. Antioxidant treatments, including resveratrol and other nutritional supplements, combined with moderate aerobic exercise, may help mitigate the clinical damage caused by oxidative stress. However, more research is required to assess the true effectiveness of these interventions [70]. A promising therapy is using stem cells to regenerate damaged valve tissue and restore normal function [73]. Also, targeting specific genetic pathways involved in valve aging and degeneration is a promising new approach [74].

Mitral valve aging involves several structural and functional changes that can significantly affect cardiovascular health. Careful monitoring and management of mitral valve health is essential to prevent severe complications in the older population.

## 6. Conclusions

The aging process of the mitral valve is complex and multifaceted, influenced by various factors including wear and tear, fibrosis, calcification, and mechanical stresses. As people age, constant exposure to mechanical stress can lead to thinning of the mitral valve leaflets, with the loss of flexibility. These processes can lead to structural changes within the valve leaflets and supporting structures, ultimately affecting valve function. While age-related alterations such as calcification are more pronounced in older individuals, the mechanical property of the valve leaflets may show minimal changes in some cases. These changes can compromise the efficiency of valve function, increasing the risk of chronic mitral regurgitation and ultimately contributing to left ventricular remodeling and heart failure. Understanding the mechanisms underlying mitral valve aging is essential for improving diagnostic and therapeutic approaches to mitigate age-related valve dysfunction and the associated cardiovascular complications.

## Data Availability

Not applicable.
